# Association of hypertensive disorders of pregnancy and gestational diabetes mellitus with developing severe retinopathy of prematurity

**DOI:** 10.1186/s40942-025-00635-y

**Published:** 2025-04-28

**Authors:** Narges Khoshtinat, Maryam Moayeri, Hanieh Fakhredin, Afsar Dastjani Farahani, Fariba Ghassemi, Alireza Mahmoudi, Fatemeh Bazvand, Amin Nabavi

**Affiliations:** 1 Eye Research Center, Farabi Eye Hospital, Tehran University of Medical Sciences, Farabi Hospital, Tehran, Iran; 2 Department of Ophthalmology, University of California Los Angeles David Geffen School of Medicine, Los Angeles, CA USA; 3 Department of Ophthalmology, University of Tennessee Health Science Center, Memphis, TN USA

**Keywords:** Hypertensive disorders of pregnancy, Gestational diabetes disorder, Retinopathy prematurity, Perinatal risk factors

## Abstract

**Purpose:**

To evaluate the impact of hypertensive disorders of pregnancy (HDP), gestational diabetes mellitus (GDM), and their combined presence (GDM-HDP) as perinatal risk factors for severe retinopathy of prematurity (ROP).

**Methods:**

The hospital records of all premature infants referred to a tertiary center between 2020 and 2022 were retrospectively reviewed. Infants born to mothers with GDM, HDP, or both were selected for analysis. Demographic variables, perinatal risk factors, as well as clinical and treatment characteristics of the infants were collected and analyzed.

**Results:**

A total of 1161 infants and 2322 eyes, including HDP group (1110 eyes from 555 infants), GDM group (944 eyes from 472 infants), and GDM-HDP group (268 eyes from 134 infants), were enrolled. The mean gestational age (GA) and birth weight (BW) for all infants were 31.6 ± 2.5 weeks and 1572 ± 427 g, respectively. A higher percentage of eyes (76.5%) in the GDM group were classified as ROP or incomplete vascularization compared to the HDP group (71.6%), with the prevalence of severe ROP also higher in the GDM group (13.5%) than in the HDP group (9.9%; *P* < 0.05 for both comparisons). The rates of ROP and severe ROP were similar between the GDM and GDM-HDP groups. When considering only infants with BW < 1500, the GDM group still exhibited a higher rate of ROP and severe ROP compared to the HDP group. Treatment was required in 17.5% of eyes in the GDM group, 16.8% in the GDM-HDP group, and 13.9% in the HDP group (*P* = 0.071).

**Conclusion:**

GDM is a higher risk factor for ROP and developing severe ROP than HDP. However, the data indicate that careful monitoring and management of both GDM and HDP during pregnancy may be crucial in mitigating the risk of severe ROP.

## Introduction

Retinopathy of prematurity (ROP) is a complex disorder characterized by abnormal retinal vascularization in premature infants. It has become the leading cause of childhood blindness worldwide. While prematurity and low birth weight are widely recognized as risk factors for ROP, numerous perinatal factors have been implicated in its development [1, 2].

Hypertensive disorders of pregnancy (HDP), including gestational hypertension, pre-eclampsia, and eclampsia, are linked to low birth weight and prematurity. However, their connection to retinopathy of prematurity (ROP) remains unclear [3]; however, the link between HDP and ROP remains inconclusive. Some studies proposed an association between HDP and increased risk of ROP development due of altered level of angiogenic factors [4–6], while others reported contrasting findings [7–9]. Maternal hyperglycemia, as another perinatal factor, can lead to fetal hyperglycemia and prematurity [10]. Furthermore, both ROP and diabetic retinopathy are associated with retinal vascular damages causing vascular leakage and neovascularization [11] which has prompted ongoing debate about connection of gestational diabetes mellitus (GDM) and ROP [12–14].

There is currently no study that compares the role of HDP and GDM on the developing severe ROP in premature infants. This study was designed to evaluate the effect of HDP in comparison with GDM, and the combined presence of GDM and HDP (GDM-HDP) as perinatal risk factors influencing the severity of ROP in premature infants.

## Methods

This retrospective study was conducted at Farabi Eye Hospital, Tehran, Iran. The local ethical committee of Tehran University of Medical Sciences approved (IR.TUMS.FARABIH.REC.1402.006) the study protocol, and the tenets of Helsinki Declaration were followed.

The medical records for all infants screened for ROP from August 2020 to December 2022 were reviewed. The screening criteria encompassed infants with a birth weight (BW) of 1500 g (g) or less or a gestational age (GA) of 30 weeks or less, along with selected infants weighting between 1500 g and 2500 or having GA of 30 to 34 weeks with an unstable clinical course referred by their attending neonatologist. Infants born to mothers with HDP, GDM, or both were eligible for inclusion in the study. However, infants whose mothers had additional risk factors beyond HDP and GDM, such as preexisting hypertension or diabetes, other serious systemic diseases, or a history of COVID-19 infection in the last trimester, were excluded from the analysis. Furthermore, infants with incomplete records also excluded. The screening protocol followed recommendations from the American Academy of Pediatrics, the American Association for Pediatric Ophthalmology and Strabismus, and the American Academy of Ophthalmology [15]. Dilated fundus examinations were conducted using a binocular indirect ophthalmoscope, a scleral depressor, and a 20-diopter condensing lens. The zone and severity of ROP were determined based on International Classification of Retinopathy of Prematurity (ICROP) [16]. Eyes were categorized as having ROP if exhibited ROP of stage 1 or higher. Severe ROP was defined as stage 3 or beyond in any retinal zone [14].

The criteria for treatment included patients whose retinopathy met the type 1 ROP criteria proposed by the BEAT-ROP study [17]. In general, eyes in zone 1 or posterior zone 2 were treated with intravitreal bevacizumab (IVB), whereas those in anterior zone 2 received either IVB or laser photocoagulation treatment. Eyes diagnosed with stage 4a ROP underwent either scleral buckling or initial laser treatment. For stage 4b and 5 ROP, lens-sparing vitrectomy or lensectomy and vitrectomy were typically employed as the treatment approach.

Demographic information and risk factors including BW, GA, gender, maternal age, singleton or multiple gestations, sepsis, intra-ventricular hemorrhage (IVH), oxygen therapy, mechanical ventilation, blood transfusion, and phototherapy were extracted from the infant’s medical records.

Data were presented using mean and standard deviation, or median and range for quantitative data, and number and percentage for qualitative data. To compare variables between groups, we used Analysis of Variance, Kruskall-Wallis test, Chi-Square test, and Fisher exact test, whenever appropriates. Generalized Estimating Equation (GEE) analysis was employed to obtain the adjusted and unadjusted odds ratio (OR) for possible confounders. Statistical analysis was performed using SPSS software for Windows, version 21 (SPSS Inc., Chicago, IL). A P value less than 0.05 was considered statistically significant.

## Results

During study period, 1181 infants of mothers of either GDM or HDP or both, visited in our clinic. Twenty infants were excluded because of incomplete baseline or follow up data. A total 1161 infants and 2322 eyes, including HDP group (1110 eyes from 555 infants), GDM group (944 eyes from 472 infants) and GDM-HDP group (268 eyes from 134 infants) were recruited in this study. There were 381 infants from multiple gestations in our study population. The mean GA and BW for all infants were 31.6 ± 2.5 (24 to 39) weeks and 1572 ± 427 (530 to 3850) g, respectively. The median follow up time was 3 (0 to 116) weeks and the median visits for each infant was 2 [1–21] times.

The demographic information and risk factors in study groups were shown in Table 1. Infants in GDM group exhibit significantly higher BW and lower GA compared to both the HDP and GDM-HDP groups (*P* < 0.05). Additionally, there are significant differences in the history of IVH (*P* = 0.015) and the utilization of phototherapy (< 0.001) across the study groups.

**Table 1:** Demographic and risk factors among study groups

		HDP (555 infants)	GDM (472 infants)	GDM-HDP (134 infants)	*P* Value
Birth weight	≤ 1500 g	289 (52.1%)	188 (39.8%)	62 (46.3%)	< 0.001
	1501 + Grams	266 (47.9%)	284 (60.2%)	72 (53.7%)	
Gestational age	≤ 30 Weeks	154 (27.7%)	165 (35%)	38 (28.4%)	0.001
	> 30 Weeks	401 (72.3%)	307 (65.0%)	96 (71.6%)	
Maternal age	Mean ± SD	32.8 ± 6.5	31.8 ± 6	35.4 ± 5.9	< 0.001
Sepsis ^a^	+	45 (8.1%)	46 (9.8%)	14 (10.4%)	0.300
	-	509 (91.9%)	424 (90.2%)	120 (89.6%)	
Intraventricular hemorrhage ^b^	+	17 (3.1%)	12 (2.6%)	0	0.015
	-	536 (96.9%)	457 (97.4%)	134	
History of oxygen therapy ^c^	+	473 (85.4%)	417 (88.5%)	114 (85.1%)	0.082
	-	81 (14.6%)	54 (11.5%)	20 (14.9%)	
History of mechanical ventilation ^c^	+	136 (24.6%)	121 (25.6%)	33 (24.6%)	0.852
	-	417 (75.4%)	351 (74.4%)	101 (75.4%)	
History of transfusion ^c^	+	132 (23.8%)	109 (23.1%)	24 (17.9%)	0.113
	-	422 (76.2%)	362 (76.9%)	110 (82.1%)	
History of phototherapy ^c^	+	290 (52.3%)	284 (60.4%)	61 (45.5%)	< 0.001
	-	265 (47.7%)	186 (39.6%)	73 (54.5%)	

HDP: Hypertensive disorders of pregnancy; GDM: Gestational diabetes mellitus

^a^ was not recorded in 3 infants

^b^ was not recorded in 5 infants

^c^ was not recorded in 2 infants

More eyes in the GDM group (76.5%) were classified as having ROP or incomplete vascularization compared to the eyes in the HDP group (71.6%; *P* < 0.05). However, there was no significant difference between the GDM-HDP and GDM groups. There were 9 eyes with stage 4a ROP, one eye with stage 4b ROP, and 13 eyes with stage 5 ROP at worst visit in the entire study groups. Among these, only 3 eyes were initially diagnosed at stage 3 in our center and progressed after treatment, while the others were initially visited or referred from other centers with stage 4 or 5. There was also a significant difference between the study groups in terms of ROP zone, ROP stage, and ROP plus at the worst examination (Table 2).

**Table 2:** Clinical characteristics among study groups

		HDP (1110 Eyes)	GDM (944 Eyes)	GDM-HDP (268 Eyes)	*P* Value
ROP presence	ROP or incomplete vascularization	795 (71.6%)	722 (76.5%)	208 (77.6%)	0.018
	No ROP	315 (28.4%)	222 (23.5%)	60 (22.4%)	
ROP zone	Complete vascularization	315 (28.4%)	222 (23.5%)	60 (22.4%)	0.016
	Zone 1	14 (1.3%)	19 (2%)	6 (2.2%)	
	Zone 2	310 (27.9%)	310 (32.8%)	72 (26.9%)	
	Zone 3	471 (42.4%)	393 (41.6%)	130 (48.5%)	
ROP stage in worst visit	No ROP	315 (28.4%)	222 (23.5%)	60 (22.4%)	0.002
	Incomplete vascularization	233 (21%)	168 (17.8%)	32 (11.9%)	
	Stage 1	274 (24.7%)	272 (28.8%)	85 (31.7%)	
	Stage 2	178 (16%)	155 (16.4%)	54 (20.1%)	
	Stage 3	101 (9.1%)	115 (12.2%)	35 (13.1%)	
	Stage 4	3 (0.3%)	7 (0.7%)	0	
	Stage 5	6 (0.5%)	5 (0.5%)	2 (0.7%)	
ROP plus in worst visit	No Plus	905 (81.5%)	749 (79.3%)	212 (79.1%)	< 0.001
	Pre Plus	138 (12.4%)	83 (8.8%)	27 (10.1%)	
	Plus	67 (6%)	112 (11.9%)	29 (10.8%)	
Severe ROP	+	110 (9.9%)	127 (13.5%)	37 (13.8%)	0.026
	-	1000 (90.1%)	817 (86.5%)	231 (86.2%)	
Initial treatment	IVB	103 (9.3%)	107 (11.3%)	29 (10.8%)	
	Laser therapy	45 (4.1%)	54 (5.7%)	14 (5.2%)	
	Surgery	6 (0.5%)	4 (0.4%)	2 (0.7%)	
	Total	154 (13.9%)	165 (17.5%)	45 (16.8%)	0.071
Retreatment after laser or IVB	+	9/148 (6.1%)	19/161 (11.8%)	6/43 (14%)	0.056
	-	139/148 (93.9%)	142/161 (88.2%)	37/43 (86%)	

HDP: Hypertensive disorders of pregnancy; GDM: Gestational diabetes mellitus; ROP: Retinopathy of prematurity; IVB: Intravitreal bevacizumab

Severe ROP was more prevalent in the GDM (13.5%) and GDM-HDP (13.8%) groups compared to the HDP (9.9%) group (*P* = 0.026). Overall, 364 eyes (15.7%) across all study groups required treatment. However, considering only infants with BW < 1500, 28% (302/1078) eyes needed interventions. The initial treatments included 239 eyes with IVB injections, 113 eyes with laser, and 12 surgeries (Scleral buckling or pars plana vitrectomy). Treatment was needed in 17.5% of eyes in the GDM group, 16.8% in the GDM-HDP group, and 13.9% in the HDP group (*P* = 0.071). In eyes underwent IVB injection or laser therapy, retreatment was necessary in 19 out of 161 (11.8%) eyes in the GDM group, 6 out of 43 (14%) in the HDP-GDM group, and 9 out of 148 (6.1%) in the HDP group; however, the difference was not statistically significant (*P* = 0.054). Eventually, three eyes (2 in the GDM groups and 1 in the HDP group) that underwent laser or IVB as the initial treatment required surgical intervention.

Considering only infants with BW < 1500, GDM group have high rates of severe ROP and treatment requirement than HDP group. Retreatment rate was also higher among infants of mothers with GDM than HDP in this age group (Table 3). After adjusting for GA, and BW, GDM was associated increased rate of ROP compared to HDP group.

**Table 3:** Clinical and treatment characteristics among study groups based on birth weight and gestational age

	Birth weight														Gestational age				
	<=1500 gr			*P*		1501 + gr			*P*		<=30 w			*P*		31 + wP			*P*
	HDP (578 eyes)	GDM (376 eyes)	GDM-HDP (124 eyes)			HDP (532 eyes)	GDM (588 eyes)	GDM-HDP (144 eyes)			HDP (308 eyes)	GDM (330 eyes)	GDM-HDP (76 eyes)			HDP (802 eyes)	GDM (614 eyes)	GDM-HDP (192 eyes)	
Severe ROP	100 (17.3%)	103 (27.4%)	24 (19.4%)	0.001		10 (1.9%)	24 (4.2%)	13 (9.0%)	< 0.001		79 (25.6%)	112 (33.9%)	25 (32.9%)	0.065		31 (3.9%)	15 (2.4%)	12 (6.3%)	0.041
Treatment requirement	141 (24.4%)	131 (34.8%)	30 (24.2%)	0.001		13 (2.4%)	34 (6.0%)	15 (10.4%)	< 0.001		111 (36%)	144 (43.6%)	33 (43.4%)	0.125		43 (5.4%)	21 (3.4%)	12 (6.3%)	0.133
Retreatment after IVB or laser	9/135 (6.7%)	17/129 (13.2%)	6/28 (21.4%)	0.042		0/13	2/32 (6.3%)	0/15	0.404		5/107(4.7%)	19/140 (13.6%)	6/31 (19.4%)	0.022		4/41 (9.8%)	0/21	0/12	0.182

HDP: Hypertensive disorders of pregnancy; GDM: Gestational diabetes mellitus; ROP: Retinopathy of prematurity; IVB: Intravitreal bevacizumab

## Discussion

This study compared the impact of GDM and HDP on ROP in premature infants. Infants born to mothers with GDM, with or without HDP, had higher rates of ROP and severe ROP than those born to mothers with HDP alone. Furthermore, very low birth weight infants belonging to the GDM group were more frequently in need of treatment for ROP.

While our study lacks a designated control group, the comparison of risks for ROP and severe ROP among HDP, GDM and the combined GDM-HDP groups has yielded insightful observations. Notably, the risk of both ROP and severe ROP appears comparable between the GDM and GDM-HDP groups; however, both GDM and GDM-HDP groups exhibit a higher risk when contrasted with the HDP group. While caution is warranted in drawing definitive conclusions without a control group, these findings imply a potential unique influence of GDM in contributing to the heightened risk of ROP and severe ROP, surpassing that associated with hypertensive disorders alone.

There is a growing body of evidence highlighting a robust association between GDM and the presence and severity of ROP. Tunay et al. [13] Focused on infants with a birth weight of 1500 g or more and observed a substantial correlation between maternal diabetes and ROP development, revealing a 25-fold increase in the overall risk of ROP and a 6.3-fold increase in the risk of type 1 ROP. Opara et al. [14] identified GDM as an independent risk factor for clinically significant ROP in infants, focusing specifically on those with a birth weight less than 1500 g. Moreover, the duration and magnitude of hyperglycemia have been implicated, with each additional day of hyperglycemia associated with an elevated risk of ROP [18]. Several hypotheses have been proposed to elucidate these findings, including the induction of vascular endothelial growth factor (VEGF) in retinal cells in culture mediums due to hyperglycemia, a phenomenon also supported by animal studies [13, 19].

On the contrary, the role of maternal HDP in the development of ROP in infants remains a topic of conflicting perspectives. Filho and colleagues [7] proposed that maternal pre-eclampsia may play a protective role against ROP. Similarly, Yu et al. [8] identified a favorable effect of pre-eclampsia on ROP incidence. In contrast some studies reported an increased risk of ROP associated with pre-eclampsia [4, 6]. Intriguingly, Shulman et al. found a correlation between pre-eclampsia and an elevated risk of ROP in an unrestricted group (without considering prematurity and low birth weight), whereas pre-eclampsia appeared protective in a restricted sub-cohort of preterm and very low birth weight infants [20]. This heterogeneity in these findings may be attributed to varying adjustments for confounding factors such as BW, GA, and others. Additionally, differences in neonatal and obstetric practices across countries, as well as variations in the definition and inclusion criteria for HDP, could contribute to this heterogeneity. Finally, a recent systematic review did not find a significant role for pre-eclampsia or any type of HDP in the incidence of ROP and severe ROP [21]. 

As the impact of HDP on ROP remains a subject of controversy, various mechanisms being proposed. Some studies have suggested that maintaining insulin-like growth factor-1 (IGF-1) levels above a certain threshold is crucial for normal angiogenesis, and alterations in these levels in the context of HDP may contribute to the development of ROP [22, 23]. Additionally, the presence of anti-angiogenic factors, such as fms-like tyrosine kinase (sFlt-1), a soluble splice variant of vascular endothelial growth factor receptor, has been observed at elevated levels in the cord blood of pregnancies affected by pre-eclampsia [24]. The dual nature of this phenomenon has been proposed, suggesting that it may act both pathogenically and potentially as a protective factor against the severe form of ROP. Some even proposed that intrauterine stress that facilitates the maturation of neonatal retinal vasculature, similar to mechanism that has been postulated for accelerated maturation and disappearance of tunica vasculosa lentis in chronic intrauterine stress [25].

Few factors have been relatively established as contributors to treatment failure in infants with ROP, including treatment modality (laser or anti-VEGF) and Zone of ROP [26, 27]. However, the role of maternal risk factors in treatment failure and the need for additional treatment is less understood. In this study, the overall retreatment rate was 9.7% among all infants, a figure comparable to large cohort studies [28, 29] that do not restrict inclusion to GDM or HDP. Nevertheless, the retreatment rate was notably higher in the GDM group compared to the HDP group, although the difference was marginally significant. This finding bears clinical significance, underscoring the importance of thorough follow-up after initial treatment in infants born to mothers with GDM.

Our study possesses several strengths when compared to similar investigations exploring the influence of GDM or GHD on the risk of ROP. Unlike some studies that rely on international classification of disease (ICD) codes for the diagnosis of GDM or GHD, we conducted a thorough evaluation of medical records, enhancing the accuracy of our findings. Furthermore, our inclusion of a diverse range of infants undergoing ROP screening, rather than restricting it solely to very low birth weight infants, contributes to the generalizability of our results. The substantial sample size further strengthens the statistical power of our analysis.

However, several limitations of our study must be acknowledged. The most significant limitation is the lack of a formal control group consisting of infants born to mothers without HDP or GDM. This absence restricts our ability to establish definitive causal relationships and compare the risk posed by these conditions to baseline risk levels in the general population. The retrospective, single-center design of our study is a crucial consideration that may impact the generalizability of our findings to broader populations. Not capturing additional maternal risk factors such as smoking is another limitation that could potentially introduce confounding variables. Additionally, our enrollment criteria focusing on infants referred for ROP examination may introduce a selection bias. To address these limitations and gain a more comprehensive understanding, continued investigation through prospective cohort studies is needed.

GDM poses a higher risk for the development of ROP and severe ROP compared to HDP. However, both conditions are associated with significant ROP-related complications, underscoring the importance of proactive prenatal care and timely neonatal screening to mitigate the risk of severe ROP and improve neonatal outcomes.

## Data Availability

No datasets were generated or analysed during the current study.
